# Rich-club organization of whole-brain spatio-temporal multilayer functional connectivity networks

**DOI:** 10.3389/fnins.2024.1405734

**Published:** 2024-05-24

**Authors:** Jianhui Zheng, Yuhao Cheng, Xi Wu, Xiaojie Li, Ying Fu, Zhipeng Yang

**Affiliations:** ^1^College of Electronic Engineering, Chengdu University of Information Technology, Chengdu, China; ^2^Huaxi Molecular Imaging Research Laboratory, Department of Radiology, West China Hospital, Sichuan University, Chengdu, China; ^3^Department of Computer Science, Chengdu University of Information Technology, Chengdu, China

**Keywords:** functional connectivity network, functional MRI, rich-club, intraclass correlation coefficient, statistical significance

## Abstract

**Objective:**

In this work, we propose a novel method for constructing whole-brain spatio-temporal multilayer functional connectivity networks (FCNs) and four innovative rich-club metrics.

**Methods:**

Spatio-temporal multilayer FCNs achieve a high-order representation of the spatio-temporal dynamic characteristics of brain networks by combining the sliding time window method with graph theory and hypergraph theory. The four proposed rich-club scales are based on the dynamic changes in rich-club node identity, providing a parameterized description of the topological dynamic characteristics of brain networks from both temporal and spatial perspectives. The proposed method was validated in three independent differential analysis experiments: male–female gender difference analysis, analysis of abnormality in patients with autism spectrum disorders (ASD), and individual difference analysis.

**Results:**

The proposed method yielded results consistent with previous relevant studies and revealed some innovative findings. For instance, the dynamic topological characteristics of specific white matter regions effectively reflected individual differences. The increased abnormality in internal functional connectivity within the basal ganglia may be a contributing factor to the occurrence of repetitive or restrictive behaviors in ASD patients.

**Conclusion:**

The proposed methodology provides an efficacious approach for constructing whole-brain spatio-temporal multilayer FCNs and conducting analysis of their dynamic topological structures. The dynamic topological characteristics of spatio-temporal multilayer FCNs may offer new insights into physiological variations and pathological abnormalities in neuroscience.

## 1 Introduction

Functional connectivity network (FCN) based on functional magnetic resonance imaging (fMRI) is established by evaluating the time-domain correlation among blood oxygen level-dependent (BOLD) signals across diverse brain regions. Since the concept of functional connectivity (FC) was introduced (Biswal et al., 1995), constructing FCN to investigate the connectivity patterns of different brain regions [motor network (Biswal et al., 1995); default mode network (Greicius et al., 2003); attention network (Fox et al., 2006)], as well as the physiological [gender (Zhou, 2016); age (Zhang et al., 2018b); fluid intelligence (Finn et al., 2015)] and pathological [autism spectrum disorder (ASD; Liu M. et al., 2021); Alzheimer’s disease (AD; Gao et al., 2020); schizophrenia (Yang C. et al., 2023)] influences on brain networks, has become one of the primary research directions in the field of fMRI.

Presently, most investigations on resting-state fMRI (rs-fMRI) brain functional networks have focused on constructing single-layer FCN based on either gray matter (GM) or white matter (WM) brain regions(Cai et al., 2021), using multiple runs of rs-fMRI data, Zhang et al. demonstrated the reliability of gender prediction through GM FCN constructed from rs-fMRI FC (Zhang et al., 2018b). Zhang et al. employed fMRI data acquired under natural viewing conditions to measure GM FC, obtaining more reliable test–retest results compared to those derived from rs-fMRI data (Cai et al., 2021). Peer et al. conducted clustering analysis on WM voxel-wise rs-fMRI data, confirming the presence of distinctive symmetric WM FCN (Peer et al., 2017).

However, the human brain is composed of multiple highly complex networks (Ames, 2000; Bullmore and Sporns, 2012). Single-layer FCN typically only reflects local brain activity and single brain functional features, underpowering to capture the intricate functional patterns between different brain regions (Hu et al., 2021). Traditional FCNs typically rely on low-order correlations and graph theory for construction, which may struggle to comprehensively describe the functional interactions between brain networks. Due to the constraints of single-layer FCN and lower-order correlations in capturing brain functional activity, researchers have endeavored to develop multilayer or high-order FCNs (Pedersen et al., 2018; Sporns, 2018; Bazinet et al., 2021; Hu et al., 2021). Most recent researches aim to capture the high temporal or spatial complexity of the human brain system and to represent the modular characteristics of brain functions. Utilizing the sliding time window method with three different dynamic FC (dFC) statistics (standard deviation, ALFF, and excursion), Zhang and colleagues explored the associations between static FC, dFC, and their reliability using intraclass correlation coefficient (ICC) (Zhang et al., 2018a). Wee and colleagues achieved enhanced individual recognition of Mild Cognitive Impairment (MCI) by integrating spatial information from whole-brain (including GM and WM) Diffusion Tensor Imaging (DTI) and rs-fMRI data (Wee et al., 2012). Yang et al. developed a novel approach for constructing high-order FCNs by integrating low-order FCNs with those constructed based on hypergraph (Yang J. et al., 2023). Wang et al. directed their focus toward the dynamic characteristics of WM BOLD signals and the reliability of dFC, substantiating the significance of WM signals in the construction of temporal dynamic FCNs (Wang et al., 2022). However, these studies have not simultaneously utilized the rich spatio-temporal dynamic features in fMRI data or have relied solely on low-order correlations for multilayer network construction.

Extensive research has been investigated on the FC features of whole-brain multilayer FCNs, but there has been a deficiency in exploring their topological characteristics. Previous studies have indicated that the brain network exhibits small-worldness, which effectively captures the dynamic and efficient information processing and transmission among brain network nodes (Achard and Bullmore, 2007; Kaiser, 2011; Yu et al., 2011; Sporns, 2013). The rich-club organization, as a crucial manifestation of small-worldness, offers a more cogent description of dynamic and diverse brain networks (Park and Friston, 2013). A large number of studies have proved that the FCNs of the human brain have the rich-club property, and the rich-club organization holds significant relevance to various cognitive and affective functions (McColgan et al., 2015; Mai et al., 2017; Zhao et al., 2021; Riedel et al., 2022). Additionally, alterations in the rich-club structure have emerged as biomarkers for studying neurological disorders, such as AD (Ma et al., 2022) and Parkinson’s disease(Liu T. et al., 2021). By analyzing the topological structure of the human brain FCN, Harriger et al. proved the existence of brain functional network hubs with high connectivity and centrality (Harriger et al., 2012). Zhou and colleagues discovered a reduction in total and global efficiency in the brain networks of ASD children through small-world characteristic analysis based on cortical thickness but not FC MRI or volumetry (Zhou et al., 2014). Van Den Heuvel and colleagues integrated rs-fMRI and DTI data to construct brain network structures, revealing and confirming the existence of rich-club organization in the resting-state brain network (van den Heuvel et al., 2009). Their another research underscores the significance of the rich-club organization in facilitating information transmission across brain regions(van den Heuvel et al., 2012). These studies, however, primarily focus on static single-layer FCNs. In recent years, the dynamic characteristics of the rich-club have gained increasing attention. Liang et al. analyzed the brain’s functional network using graph theory and provided evidence that the rich-club organization plays a role in the network’s dynamic changes(Liang et al., 2016). These studies have introduced new perspectives for investigating the rich-club organization in spatio-temporal multilayer FCNs.

In this paper, we propose a novel approach for constructing spatio-temporal whole-brain (including GM and WM) multilayer FCNs and define four metrics for analyzing the dynamic rich-club organization of proposed FCNs. Concisely, we first employed graph and hypergraph methods, along with sliding time window approach, to construct spatio-temporal multilayer FCNs. Subsequently, we employed neural network methods to preliminarily validate the efficacy of extracting spatial features from the proposed FCNs. Furthermore, we introduced four novel rich-club metrics, including temporal centrality, temporal stability, local functionality, and joint functionality, to capture the dynamic spatio-temporal characteristics of the topological structure of the brain network. Finally, the effectiveness of the proposed method was validated through three independent difference analysis tasks: male–female gender differences analysis, analysis of abnormality in patients with ASD, and individual differences analysis.

## 2 Materials and methods

### 2.1 Dataset and preprocessing

The human connectome project (HCP) dataset was employed in this study for male–female gender differences analysis and (Van Essen et al., 2012). These 1,200 Subjects Release (S1200) includes behavioral and 3 T MRI data from 1,206 healthy young adult participants collected in 2012–2015, with the following parameters: repetition time (TR) of 720 ms, echo time (TE) of 33.1 ms, flip angle (FA) of 52°, resolution of 2.0 mm, and a matrix size of 104 × 90. Each participant underwent two resting-state sessions, with a one-day interval between data collection sessions. The preprocessed data obtained from the HCP minimal preprocessing pipeline were employed in this work, including gradient distortion correction, head motion correction, image distortion correction, spatial normalization to the standard Montreal Neurological Institute (MNI) template, and intensity normalization. Furthermore, we regressed out the covariates of head motion and cerebrospinal fluid (CSF) signal and subsequently applied a bandpass filter (0.01–0.08 Hz) to attenuate noise. We randomly selected 80 male and 80 female participants based on gender. The selected data is divided into REST1 and REST2 (two scans on different days), where the data from REST1 is used for gender difference analysis, while data from both REST1 and REST2 are used for individual difference analysis. All samples with abnormal head motion parameters and quality control (QC) scores were excluded.

The ABIDE-I public dataset was utilized for analysis of abnormality in patients with ASD (Di Martino et al., 2014). The ABIDE-I dataset, contributed by 17 international institutions, comprises 1,112 participants, consisting of 539 individuals diagnosed with ASD and 573 typically developing controls (TDC). The average age of all participants was 14.7 years. These data underwent a preprocessing pipeline similar to that of the HCP dataset, including the removal of unstable time points, temporal layer correction, head motion correction, spatial registration, spatial smoothing, bandpass filtering (0.01–0.08 Hz), and regressing out covariates of head motion and CSF signal. The participant selection principle for ASD patients and TDC data involved random selection of time series lengths greater than 150 ms, with samples meeting the quality check for phenotype data. Finally, a total of 87 ASD patients and 86 TDC were selected. Table 1 presents detailed demographic information about all subjects. DPABI software V5.4 (Yan et al., 2016)^1^ was used to all preprocessing pipeline.

**Table 1:** Demographic and clinical characteristics of HCP and ABIDE-I datasets.

Clinical phenotype	HCP		ABIDE-I	
	Male (*n* = 80)	Female (*n* = 80)	ASD (*n* = 87)	TDC (*n* = 86)
Age (Mean ± SD)	27.26 ± 2.74	30.06 ± 3.38	17.31 ± 5.07	15.36 ± 3.56
Male/Female	80/0	0/80	80/7	66/20

Two-tailed *t*-tests was employed to examine the significant age differences between different groups in the dataset: HCP: *p* < 0.001; ABIDE-I: *p* = 0.004.SD, standard deviation.

**Figure 1:**
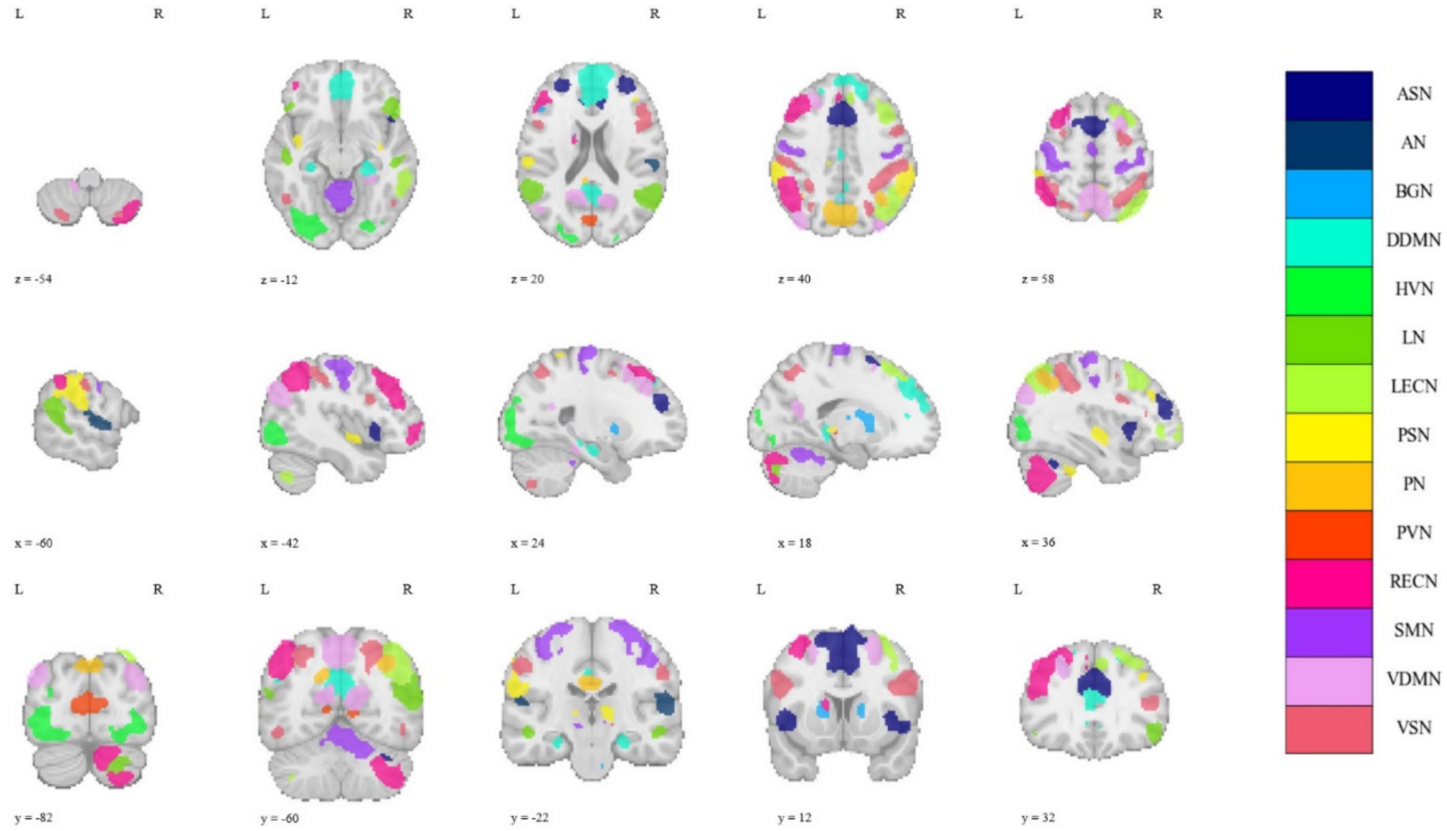
The spatial distribution of all 14 MRSNs in the brain.

The regions of interest (ROIs) division of the GM used the functional parcellation atlas provided by the Neuropsychiatric Disorders Functional Imaging Laboratory of Stanford University, which is composed of 90 sub-regions divided into 14 macroscale resting state networks (MRSNs): anterior salience network (ASN), posterior salience network (PSN), auditory network (AN), basal ganglia network (BGN), dorsal default mode network (DDMN), ventral default mode network (VDMN), primary visual network (PVN), higher visual network (HVN), language network (LN), left executive control network (LECN), right executive control network (RECN), precuneus network (PN), sensorimotor network (SMN), visuospatial network (VSN) (Shirer et al., 2012). Figure 1 illustrates the spatial distribution of the 14 MRSNs in the brain. For detailed parcellation information of the aforementioned brain GM regions, please refer to Supplementary Figure S1 and Supplementary Table S1. The ROI division of the WM used the JHU DTI-based WM atlases provided by Johns Hopkins University (Mori et al., 2008), which divided the brain WM into 48 probabilistic WM tracts. In this study, we considered the collective set of 48 WM fiber bundles as a single MRSN. Finally, at the MRSN scale, we established 15 MRSNs, comprising 14 GM MRSNs and 1 WM MRSN. At the ROI scale, we defined 138 ROIs, which consisted of 90 GM regions and 48 WM regions. Additionally, the XTRACT HCP probabilistic tract atlases were employed in the robustness validation experiments of the proposed method across different brain templates (Warrington et al., 2020). The specific information about the two WM templates can be found in Supplementary Figure S2.

### 2.2 Spatio-temporal multilayer FCNs

**Figure 2:**
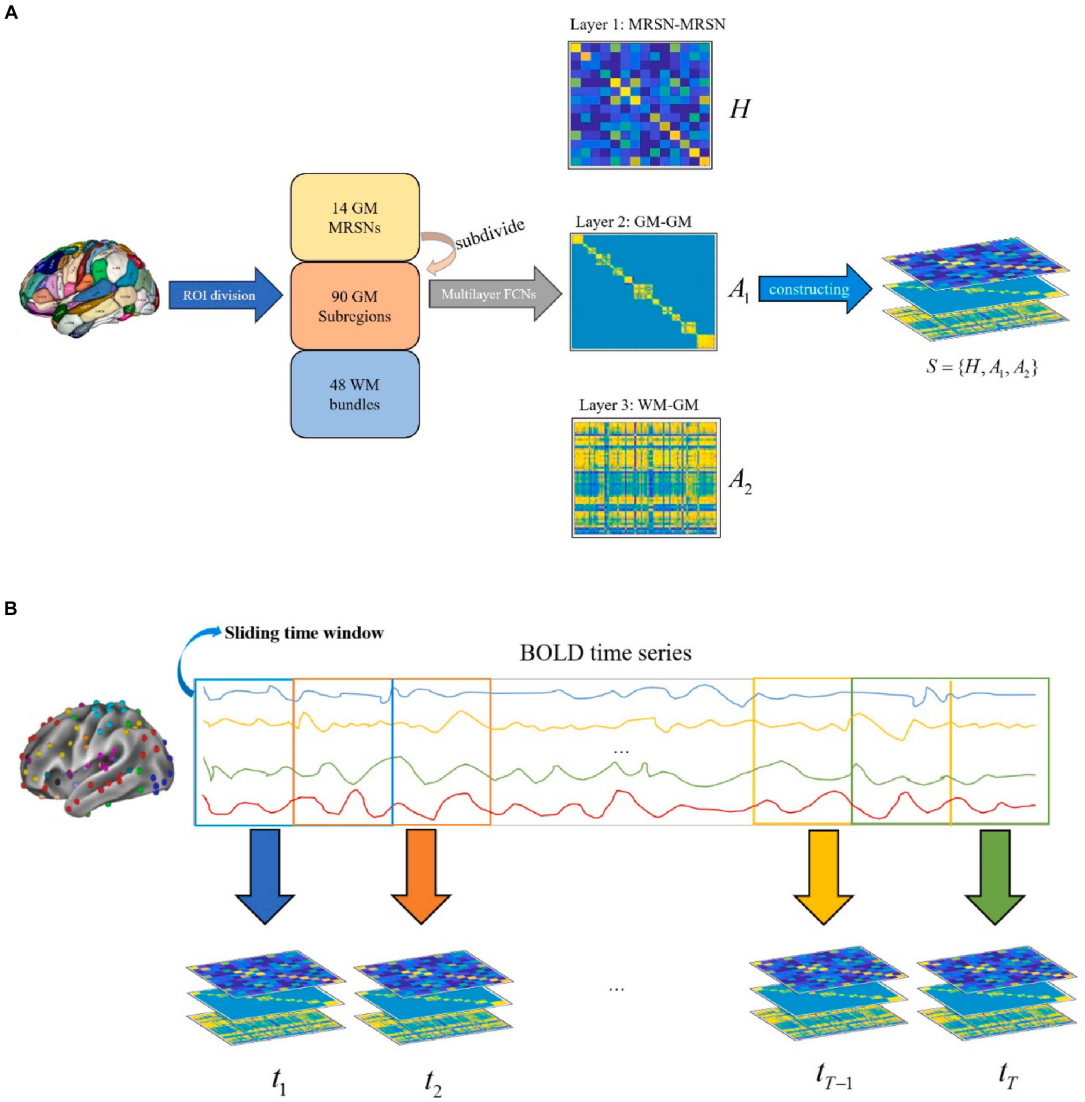
Flowchart for the construction of whole-brain spatio-temporal multilayer FCNs. **(A)** The spatial multilayer FCNs comprehensively describes the interactions between GM functional brain networks, the interactions within GM functional brain networks, and the WM-GM functional associations from a whole-brain perspective. **(B)** The temporal multilayer FCNs, based on the sliding time window method, describes the temporal dynamic characteristics of the spatial multilayer FCNs by partitioning the time series into different segments.

Figure 2A shows the specific details and flow of the spatial construction of multilayer FCNs. Considering the occurrence of coordinated functional activation across multiple brain regions in brain neural activity, we propose a novel method for constructing multilayer FCNs. This approach combines simple graphs with hypergraphs, introducing both lower-order and higher-order associations into brain FCNs simultaneously.

A hypergraph G=(ν,ε) consists of a set of nodes $\nu =\left(v_{1},v_{2},\cdots ,v_{N-1},v_{N}\right)$ with a set of hyperedges $\varepsilon =\left(e_{1},e_{2},\cdots ,e_{M-1},e_{M}\right)$ . In this work, hypergraph nodes represent brain ROIs, and hyperedges represent high-order functional interactions among these ROIs. A hypergraph can be $H\in {\mathbb{R}}^{N\times M}$ , which define as follows:


(1) $$H_{ij}=\{\begin{array}{cc} 1 & v_{i}\in e_{j}\\ 0 & otherwise \end{array}$$

In light of previous researches (Jie et al., 2016; Fan et al., 2020), we employed a representation-based approach to construct the incidence matrix H , specifically, lasso sparse linear regression was applied to estimate the incidence matrix:


(2) $$\underset{\alpha_{i}}{\min}\frac{1}{2}{\left\|X_{i}-B_{i}\alpha_{i}\right\|}_{2}^{2}+\lambda {\left\|\alpha_{i}\right\|}_{1}$$

Where $X_{i}$ represents the rs-fMRI time series corresponding to the central response vector, and $B_{i}=\left(X_{1},\;\cdots ,\;X_{i-1},\;X_{i+1},\;\cdots ,\;X_{N}\right)$ is a matrix composed of time series corresponding to all other nodes except node $v_{i}$ . $\alpha_{i}$ is the regression coefficient vector, where elements with larger values indicate a stronger association between the corresponding node and the central node $v_{i}$ . In this work, nodes with a value greater than zero in $\alpha_{i}$ are considered to belong to hyperedge $e_{i}$ . λ is the regularization coefficient, which is used to control the sparsity of the regression results.

The simple graph is represented using a threshold binary adjacency matrix A , where the threshold is determined by the mean plus one standard deviation of FC strengths. This adaptive approach filters out weak FC.

By combining pre-defined multi-scale ROIs with the proposed method, we constructed a three-layer FCN at the spatial scale, aiming to characterize the spatial structure of the whole brain (including GM and WM) from the perspectives of both low-order and high-order associations. The first layer FCN is represented by the incidence matrix H of the hypergraph. Specifically, we successively took each MRSN as a central node, applied the Lasso sparse regression algorithm to estimate 14 hyperedges, and constructed the incidence matrix $H\in {\mathbb{R}}^{14\times 14}$ of the hypergraph based on Equation (1). The first layer FCN describes the high-order associations between MRSN regions, while the second layer FCN focuses on representing the low-order associations within sub-regions of each MRSN, represented by the adjacency matrix $A_{1}\in {\mathbb{R}}^{90\times 90}$ . The third layer FCN represents the low-order associations between these GM subregions and the brain WM bundles, represented by the adjacency matrix $A_{2}\in {\mathbb{R}}^{48\times 90}$ .

For the construction of temporal multilayers, the sliding time window method is utilized to partition the whole BOLD time series signal into overlapping segments. Following this, spatial multilayer FCNs are established for each time series segment, resulting in multilayer FCNs representing distinct temporal periods. Figure 2B illustrates the procedure for constructing the temporal layers of the spatio-temporal multilayer FCNs. The length and sliding stride of the time window determined the number of temporal layers T in the multilayer FCNs, with the specific calculation detailed as follows:


(3) $$T=\frac{L-W}{S}+1$$

Where L is the length of the original BOLD time series, W is the length of the time window, and S is the sliding stride of adjacent time windows. Based on previous researches and the actual parameters of the datasets (Pedersen et al., 2018; Fan et al., 2020), we employed distinct time window parameters for two different datasets: For the HCP dataset, W =200TR, S =100TR, L =1200TR; and for the ABIDE-I dataset, W =20TR, S =10TR, L =150TR.

### 2.3 Rich-club organization

In this work, we determined the core nodes forming the rich-club organization by calculating the normalized node degree of 90 GM subregions and 48 WM regions in the multilayer FCNs. It is important to note that, for the node degree of the 90 GM subregions in the first layer FCN, we define the node degree of a GM subregion to be consistent with the node degree of its corresponding MRSN. The node degrees for both the adjacency matrix and incidence matrix can be calculated according to the Equation (4):


(4) $$\{\begin{array}{c} D\left(i\right)=\sum_{j=1,j\neq i}^{N}A_{ij}\\ D_{hyper}\left(i\right)=\sum_{e_{j}\in \varepsilon }H_{ij} \end{array}$$

Subsequently, we selected the top 15 nodes based on normalized node degree to form the core nodes constituting the rich-club organization, while considering the remaining nodes as peripheral nodes. The motivation behind selecting the top 15 nodes is derived from the pre-defined set of 15 MRSNs. The node degree normalization methods can be obtained as follows:


(5) $$D_{norm}\left(i\right)=\frac{\sum D^{l}\left(i\right)}{\sum D_{\max}^{l}\left(i\right)}$$

Where $D^{l}\left(i\right)$ is the node degree of node $v_{i}$ in layer l of the multilayer FCNs, $D_{\max}^{l}\left(i\right)$ represents the theoretical maximum node degree of node $v_{i}$ in layer l . It is important to note that the second layer FCN represents FC between subregions of each GM MRSN, and the number of subregions differs among distinct MRSNs. Consequently, there will be variations in $D_{\max}^{l}\left(i\right)$ for the 90 GM nodes. For example, for a GM node belonging to the AN (containing 3 GM nodes), the $D_{\max}$ for the first layer is 13, for the second layer is 2, and for the third layer is 48. Additionally, since the 48 WM nodes are only applied in the third layer FCN, $\sum D_{\max}^{l}\left(i\right)$ =90 in this work.

### 2.4 Rich-club metrics

The dynamic variations of rich-club organization represent a significant characteristic of functional interactions and information transfer among brain regions. Parameterized representation of the dynamic change of core brain regions allows for quantitative assessment of their importance and functional interaction tendency in brain activities. To describe the dynamic changes of the rich-club organization of spatio-temporal multilayer FCNs from both temporal and spatial perspectives, we innovatively introduced four distinctive rich-club metrics, namely temporal centrality, temporal stability, local functionality, and joint functionality. The specific definitions of these four metrics are as follows:

#### 2.4.1 Temporal centrality

The temporal dynamic characteristics of the rich-club organization result in the possibility of temporal fluctuations in the identity of each brain node, meaning that the identity of node may continuously shifts between core node and peripheral node over the entire time period. As a result, certain nodes may assume the core node status for only a limited duration during the entire time period. To evaluate the significance of node across the entire time period, we have defined the temporal centrality as a metric, and it is defined as follows:


(6) $$TC_{i}=\frac{1}{T}\sum_{t=1}^{T}R_{i}\left(t\right)$$

Where T represents the number of time layers of the entire sequential multilayer network. $R_{i}\left(t\right)$ represents the core node membership of node $v_{i}$ at time layer t . If, at temporal layer t , node $v_{i}$ belongs to the core nodes, then $R_{i}\left(t\right)$ =1; otherwise, $R_{i}\left(t\right)$ =0.

#### 2.4.2 Temporal stability

Temporal centrality provides a measure of a node’s importance in whole-brain FCNs across a global time scale. However, different nodes may exhibit distinct temporal distributions as core nodes. Some nodes might maintain their core attributes over an extended period, while others may undergo frequent shifts in their status. Thus, we introduce the concept of temporal stability, a metric designed to gauge the frequency of transitions between core and peripheral node identities over time. This aims to characterize the stability of the rich-club organization over temporal scales. The mathematical definition of temporal stability is as follows:


(7) $$TS_{i}=1-\frac{1}{T-1}\sum_{t=1}^{T}\delta \left[R_{i}\left(t\right),\;R_{i}\left(t+1\right)\right]$$

Where δ represents the node identity consistency function, where $\delta \left[R_{i}\left(t\right),R_{i}\left(t+1\right)\right]$ =1 when the identity of node $v_{i}$ is consistent between time layer t and t+1 ; otherwise, $\delta \left[R_{i}\left(t\right),R_{i}\left(t+1\right)\right]$ =0.

#### 2.4.3 Local functionality

Based on the definition of rich-club organization, the core nodes are evidently interconnected with numerous peripheral nodes in the network. These peripheral nodes can leverage shared core nodes to enhance information transmission and functional interaction between nodes. Under these conditions, there is likely to be functional similarity or correlation between these brain nodes, and the subregion nodes within each of the 15 MRSNs partitioned based on the *a priori* anatomical template exhibit functional consistency. To measure the functional interaction and communication capacity among brain nodes within different MRSNs, we have defined a metric called local functionality. This metric aims to reflect the functional consistency among nodes within the same MRSN. The mathematical definition of local functionality is as follows:


(8) $$\{\begin{array}{c} P_{ij}=\frac{1}{T*n_{R}}\sum_{t=1}^{T-1}\beta_{ij}^{t}\\ LF_{i}=\frac{1}{n_{s}}\sum_{j\in s}P_{ij} \end{array}$$

Where $n_{R}$ =15 is the number of core nodes, $\beta_{ij}^{t}$ denotes the number of core nodes to which both peripheral nodes $v_{i}$ and $v_{j}$ are concurrently connected at time layer t , $n_{S}$ represents the number of nodes in the functional network S (i.e., MRSN), $LF_{i}$ means the frequency with which node $v_{i}$ shares core nodes with other nodes belonging to the same functional network S .

#### 2.4.4 Joint functionality

Local functionality exclusively quantifies a node’s communication capability with other nodes within its corresponding MRSN. However, certain nodes might exhibit strong interactions with nodes in other MRSN regions. These nodes typically serve as bridge for functional interactions or information transmission between distinct functional networks. Therefore, we propose joint functionality as a rich-club metric. This metric aims to assess the communication capacity among nodes in different functional networks and reflect the level of functional interaction between distinct functional networks. The mathematical definition of joint functionality can be obtained as follows:


(9) $$JF_{i}=\frac{1}{N-n_{S}}\sum_{j\in S}P_{ij}$$

Where $JF_{i}$ is the frequency that node $v_{i}$ , belonging to functional network S , shares core nodes with nodes from other functional networks.

### 2.5 Statistical analysis

Four rich-club metrics were employed in three difference analysis tasks: gender differences analysis, analysis of abnormalities in patients with ASD, and individual differences analysis. Individual differences refer to significant distinctions between individual subjects within a group, allowing for the identification of specific individuals based on individual differences. Gender differences denote characteristic distinctions between two groups, male and female. Abnormalities in patients with ASD signify feature differences between ASD group and TDC group.

Independent sample *t*-tests were employed for the analysis of male–female gender differences and analysis of abnormality in patients with ASD. For the gender difference feature analysis of fMRI data, a total of 160 subjects were included, with 80 males and 80 females. For the analysis of abnormality in patients with ASD there were 173 participants, including 87 ASD patients and 86 TDC. The degrees of freedom for independent sample *t*-tests in both experiments were 158 and 171, respectively. All multi-tests were corrected by false discovery rate (FDR).

ICC were employed to assess the test–retest reliability of rich-club metrics for each ROI in the rs-fMRI data collected from two-scans on different days (160 REST1 data and 160 REST2 data). If a specific ROI exhibits high test–retest reliability of rich-club metrics for the same subject, this would be evidencing the presence of individual differences between participants across the two scanning sessions. The mathematical definition of ICC is as follows:


(10) $$ICC=\frac{\sigma_{r}^{2}-\sigma_{w}^{2}}{\sigma_{r}^{2}+\left(n-1\right)\sigma_{w}^{2}}$$

Where $\sigma_{r}^{2}$ is the variance of the parameter among individuals, $\sigma_{w}^{2}$ is the variance between the two-scan data for an individual, n denotes the number of data values per group. Considering previous research and the weaker nature of WM signals (Koo and Li, 2016), we employed different ICC criteria for GM and WM regions: for a GM region, if ICC is greater than 0.6, it is considered to reflect individual differences; for WM regions, an ICC greater than 0.5 is considered to reflect individual differences. For all brain regions, if the ICC is greater than 0.8, it is considered that the region significantly reflects individual differences.

## 3 Results

This work primarily utilizes whole-brain multilayer FCNs and four rich-club metrics to analyze significant differences among different groups. Specifically, we initially employed neural networks to preliminarily validate the effectiveness of the proposed whole-brain multilayer FCNs in extracting structural features. Subsequently, we conducted male–female gender differences analysis, analysis of abnormality in patients with ASD and individual differences analysis for the four rich-club metrics at both whole-brain scale and MRSN scale. Simultaneously, in the analysis of individual differences, we further investigated the topological characteristics of the WM at the ROIs scale.

The whole-brain scale rich-club metrics were obtained by averaging the corresponding metric values across all ROIs. In contrast, the MRSN scale rich-club metrics were obtained by averaging the rich-club metric values of ROIs within the corresponding sub-regions. It is important to note that since temporal centrality directly evaluate the relative importance of each ROI within the whole-brain, the values of temporal centrality for all ROIs are always equal to 1 for each subject. Therefore, in all experiments, we did not conduct an analysis of temporal centrality at the whole-brain scale.

### 3.1 The effectiveness of multilayer FCNs in spatial feature extraction

In previous studies, the sliding time window method has been widely employed to investigate the temporal dynamics of brain networks. To assess the efficacy of the proposed FCNs structure in spatial feature extraction, we employed the ConvLSTM model for preliminary validation. ConvLSTM, a classic neural network model combining conventional neural network (CNN) and recurrent neural network (RNN) (Shi et al., 2015), is proficient in handling sequential image data, aligning well with our proposed model. Specifically, we designed a ConvLSTM model with a multimodal CNN for the spatio-temporal multilayer FCNs. Given the disparate shapes of multilayer FCNs, distinct CNN architectures were devised for each layer to learn low-dimensional embeddings of structural features from FCNs with different shapes. Subsequently, mirroring the third layer of multilayer FCNs, we employed a similar approach to construct a traditional single-layer FCN (comprising 90 GM ROIs and 48 WM ROIs) as a control. Finally, with respect to the dataset comprised of two distinct methods for constructing FCN, we inputted them into the ConvLSTM model and conducted three classification experiments.: individual identification, gender classification, and ASD patient classification. It is noteworthy that the ConvLSTM model applied to the traditional single-layer FCN utilizes a single-modal CNN, consistent with the CNN model employed in the third layer of the multi-modal CNN.

**Figure 3:**
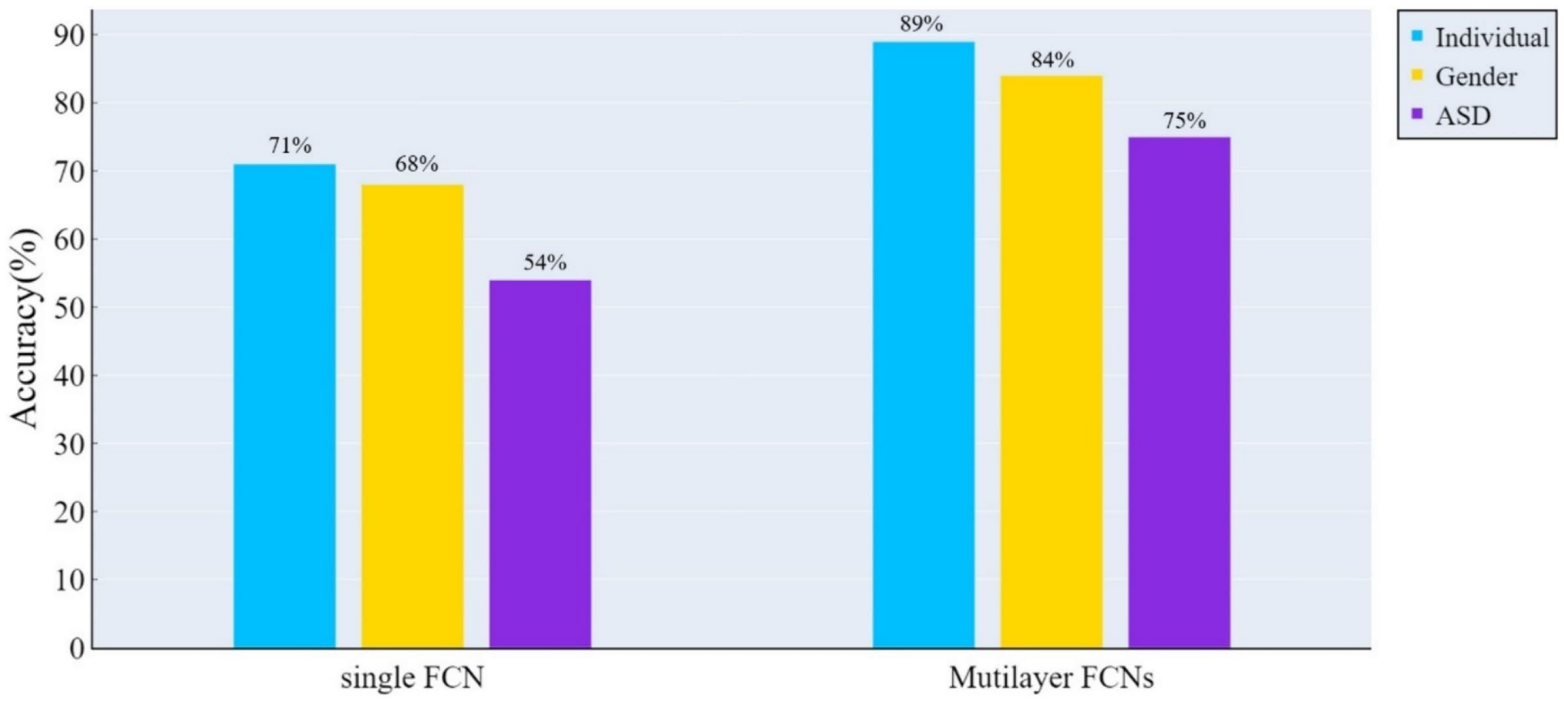
The accuracy of classification tests was assessed using the ConvLSTM model on both the traditional single-layer FCN and spatio-temporal multilayer FCNs. The results indicate that spatio-temporal multilayer FCNs achieved higher accuracy in all classification tasks.

Figure 3 illustrates the performance of the two FCN architectures across various classification tasks. The results indicate that multilayer FCNs consistently exhibit higher classification accuracy in all tasks.

### 3.2 Prominent rich-club metrics in the whole brain

Temporal centrality and temporal stability define core brain nodes across the entire time dimension from two perspectives: the frequency of being core nodes and the duration of maintaining core status. On the other hand, local functionality and integrated functionality define core nodes as bridges for functional interactions, characterizing the inclination of whole-brain nodes to engage in functional interactions. Brain nodes with higher rich-club metrics values exhibit representativeness in their corresponding feature space. In this work, we calculated the average values of these four rich-club metrics for all typically developing subjects (excluding ASD patients) and ranked them separately.

Tables 2 and 3 display highlighted ROIs information for time-related and function-related rich-club metrics, respectively. The study results indicate that 7 ROIs simultaneously exhibit high temporal centrality and stability, while 5 ROIs simultaneously demonstrate high local functionality and joint functionality. Among these regions, 3 ROIs consistently show high values across all rich-club metrics. These findings suggest a higher consistency in temporal centrality and stability, a point that is further validated in subsequent experiments. Additionally, we explored the correlation between time-related and function-related rich-club metrics. Among the 7 ROIs with high time-related rich-club metrics, 6 demonstrate high local functionality, with 4 exhibiting high joint functionality. This suggests a higher consistency between local functionality and time-related rich-club metrics.

**Table 2:** Top 10 ROIs ranked by time-related rich-club metric.

Rich-club metric	Index	Value	Label
Temporal centrality	91	0.6341	Middle cerebellar peduncle
	94	0.6153	Body of corpus callosum
	124	0.5358	Sagittal stratum L
	75*	**0.5136**	**Left Precuneus**
	3*	**0.5068**	**Anterior Cingulate Cortex, Medial Prefrontal Cortex, Supplementary Motor Area**
	1*	**0.4284**	**Left Middle Frontal Gyrus**
	**81**	**0.3773**	**Left Inferior Parietal Sulcus**
	**62**	**0.3659**	**Left Crus I, Crus II, Lobule VI**
	**45**	**0.3386**	**Right Supramarginal Gyrus, Inferior Parietal Gyrus**
	**4**	**0.3221**	**Right Middle Frontal Gyrus**
Temporal stability	1*	**0.3**	**Left Middle Frontal Gyrus**
	3*	**0.2903**	**Anterior Cingulate Cortex, Medial Prefrontal Cortex, Supplementary Motor Area**
	**4**	**0.2858**	**Right Middle Frontal Gyrus**
	75*	**0.2773**	**Left Precuneus**
	**85**	**0.2631**	**Right Inferior Parietal Lobule**
	**62**	**0.2608**	**Left Crus I, Crus II, Lobule VI**
	**81**	**0.2539**	**Left Inferior Parietal Sulcus**
	16	0.2534	Medial Prefrontal Cortex, Anterior Cingulate Cortex, Orbitofrontal Cortex
	93	0.2426	Genu of corpus callosum
	95	0.2409	Splenium of corpus callosum

Bold font indicates consistently high values in time-related rich-club metrics, and *denotes consistently high values across all rich-club metrics.

**Table 3:** Top 10 ROIs ranked by function-related rich-club metric.

Rich-club metric	Index	Value	Label
Local functionality	3*	**0.3217**	**Anterior Cingulate Cortex, Medial Prefrontal Cortex, Supplementary Motor Area**
	1*	**0.3062**	**Left Middle Frontal Gyrus**
	4	0.2865	Right Middle Frontal Gyrus
	**58**	**0.2849**	**Right Middle Frontal Gyrus, Right Superior Frontal Gyrus**
	2	0.2621	Left Insula
	**81**	**0.2581**	**Left Inferior Parietal Sulcus**
	60	0.2563	Right Inferior Parietal Gyrus, Supramarginal Gyrus, Angular Gyrus
	85	0.2535	Left Crus I, Crus II, Lobule VI
	75*	**0.2525**	**Left Precuneus**
	5	0.3221	Right Insula
Joint functionality	3*	**0.2252**	**Anterior Cingulate Cortex, Medial Prefrontal Cortex, Supplementary Motor Area**
	75*	**0.2175**	**Left Precuneus**
	**58**	**0.2136**	**Right Middle Frontal Gyrus, Right Superior Frontal Gyrus**
	1*	**0.2093**	**Left Middle Frontal Gyrus**
	53	0.2089	Right Precuneus
	34	0.1963	Left Middle Frontal Gyrus, Superior Frontal Gyrus
	64	0.1957	Left Precentral Gyrus, Postcentral Gyrus
	**81**	**0.1945**	**Left Inferior Parietal Sulcus**
	16	0.1937	Medial Prefrontal Cortex, Anterior Cingulate Cortex, Orbitofrontal Cortex
	94	0.1936	Body of corpus callosum

Bold font indicates consistently high values in function-related rich-club metrics, and *denotes consistently high values across all rich-club metrics.

### 3.3 Male–female gender differences

**Figure 4:**
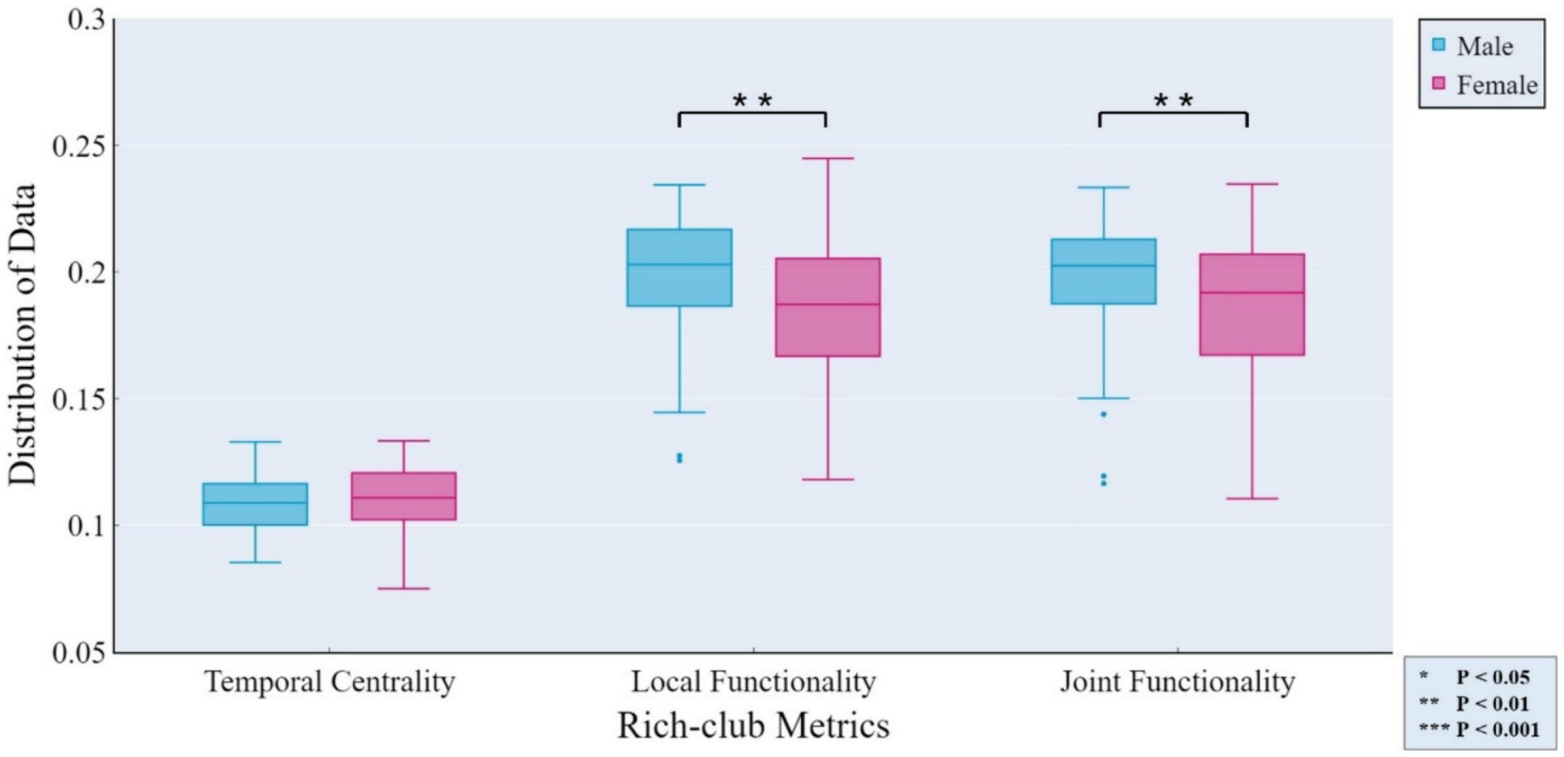
Gender differences in rich-club metrics at the whole-brain scale. The independent samples *t*-test results reveal significant differences between male and female groups in terms of local functionality and joint functionality at the whole-brain level.

The independent samples *t*-test method was employed for investigating gender differences. Specifically, we evaluated whether there were significant differences (*p* < 0.05) in rich-club metrics between male and female groups, aiming to determine whether the corresponding features could reflect group differences. Figure 4 displays the results of gender differential analysis at the whole-brain scale for temporal stability, local functionality, and joint functionality.

**Figure 5:**
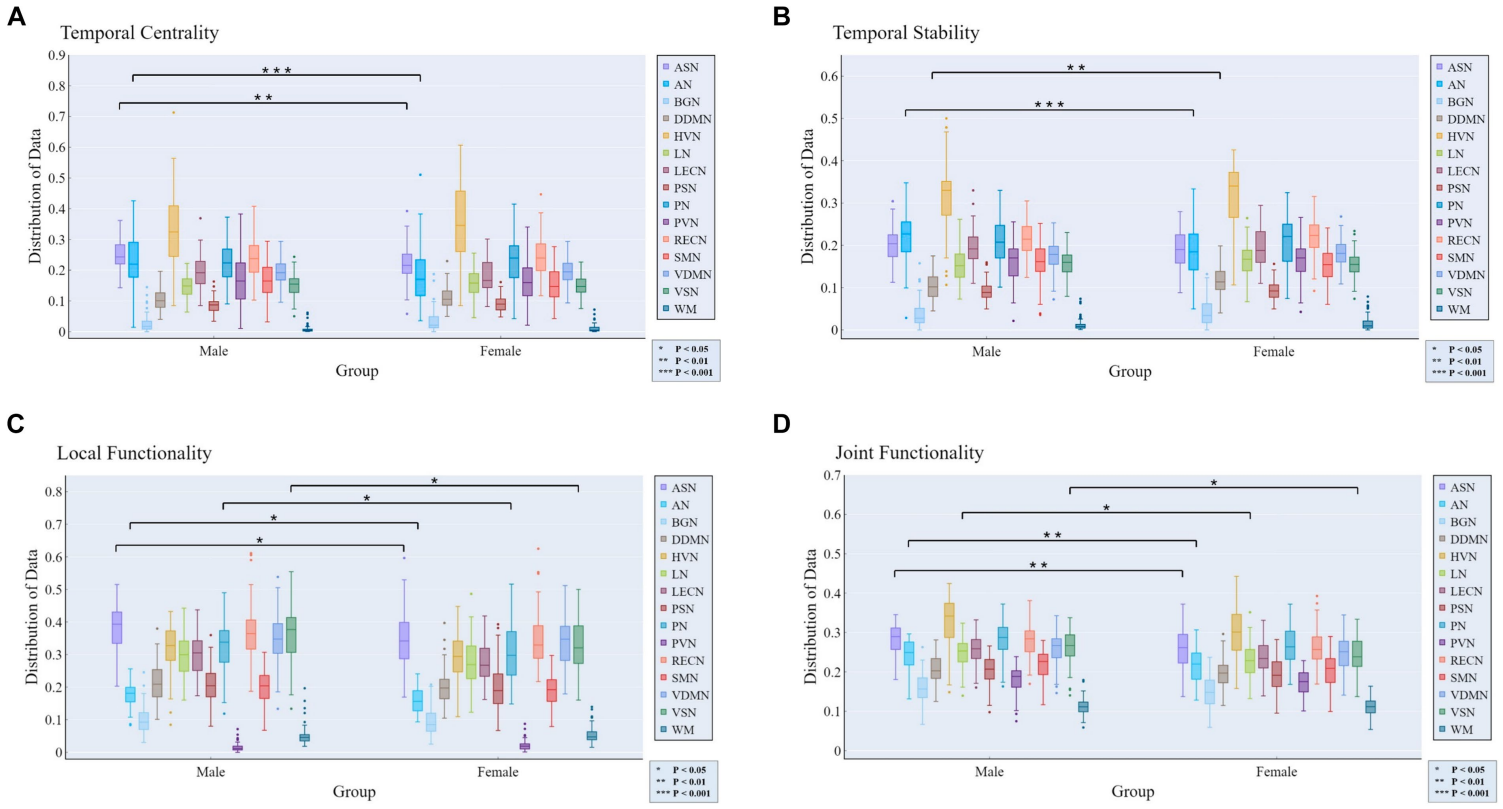
Gender differences in the four rich-club metrics at the MRSN scale. The results of independent samples *t*-tests indicate significant differences between male and female groups in all rich-club metrics at the MRSN scale. Specifically, significant differences manifest in **(A)** the temporal centrality of ASN and AN; **(B)** the temporal stability of AN and DDMN; **(C)** the local functionality of ASN, AN, PN, and VSN; and **(D)** the joint functionality of ASN, AN, LN, and VSN.

**Figure 6:**
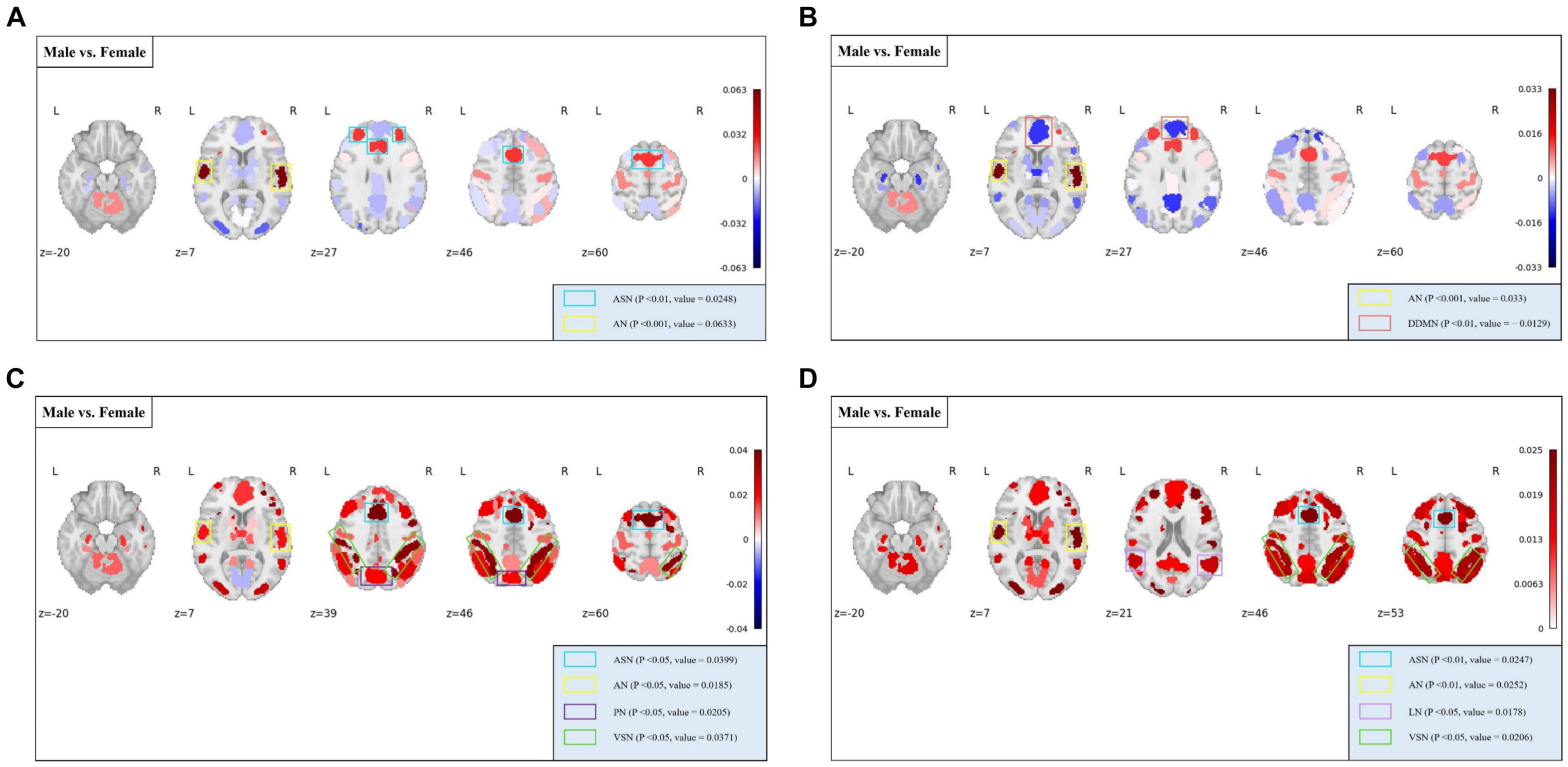
The distribution of differences in group-level average rich-club metrics between the male and female groups. Colored boxes: Indicate MRSNs with significant differences; P: *p*-value of independent samples *t*-test; value: Difference between Male and Female. **(A)** Temporal centrality; **(B)** Temporal stability; **(C)** Local functionality; **(D)** Joint functionality.

At the MRSN scale, gender differences in various functional brain regions are illustrated in Figure 5, while Figure 6 depicts the changes in male group-level average results of the proposed rich-club metrics compared to the female group. Specifically, significant differences were observed between the male and female groups in temporal centrality for ASN (M/F: 0.2476/0.2228) and AN (M/F: 0.2372/0.1739), with both showing higher values in males compared to females. Significant differences in temporal stability were found for AN (M/F: 0.2211/0.1881) and DDMN (M/F: 0.1035/0.1164), with males exhibiting significantly higher temporal stability in AN, while females showed significantly higher temporal stability in DDMN. Additionally, significant differences were observed in local functionality for ASN (M/F: 0.3826/0.3427), AN (M/F: 0.1765/0.1580), PN (M/F: 0.3271/0.3066), and VSN (M/F: 0.3684/0.3313), all of which were significantly higher in males compared to females. Similarly, significant differences were found in joint functionality for ASN (M/F: 0.2822/0.2575), AN (M/F: 0.2418/0.2166), LN (M/F: 0.2481/0.2303), and VSN (M/F: 0.2633/0.2424), all of which were significantly higher in males compared to females. Please refer to Supplementary Table S2 for the group-level average results of male and female groups for all MRSNs.

### 3.4 Abnormality in patients with ASD

For the analysis of abnormality in patients with ASD, a method similar to the gender differences analysis was employed. Specifically, we assessed whether there were significant differences (*p* < 0.05) in the rich-club metrics between the ASD patient group and the TDC group to determine whether the corresponding features could reflect the abnormalities in ASD patients.

At the whole-brain scale, temporal stability, local functionality, and joint functionality were not significantly different between the two groups of ASD patients and TDC.

**Figure 7:**
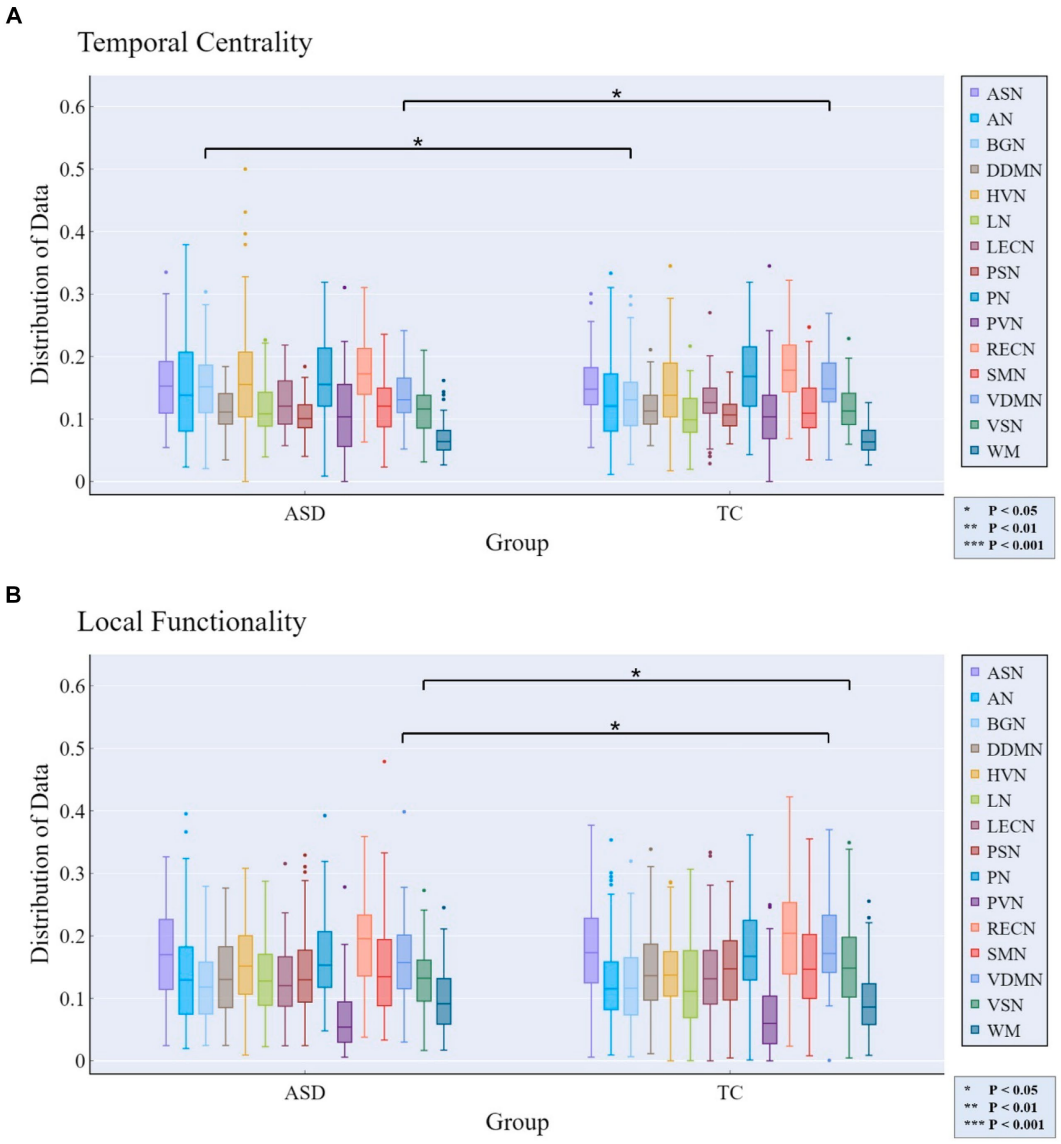
Rich-club metrics exhibit significant differences at the MRSN scale between the ASD and TDC groups. Specifically, significant differences manifest in **(A)** the temporal centrality of the BGN and VDMN; and **(B)** the local functionality of the VDMN and VSN.

**Figure 8:**
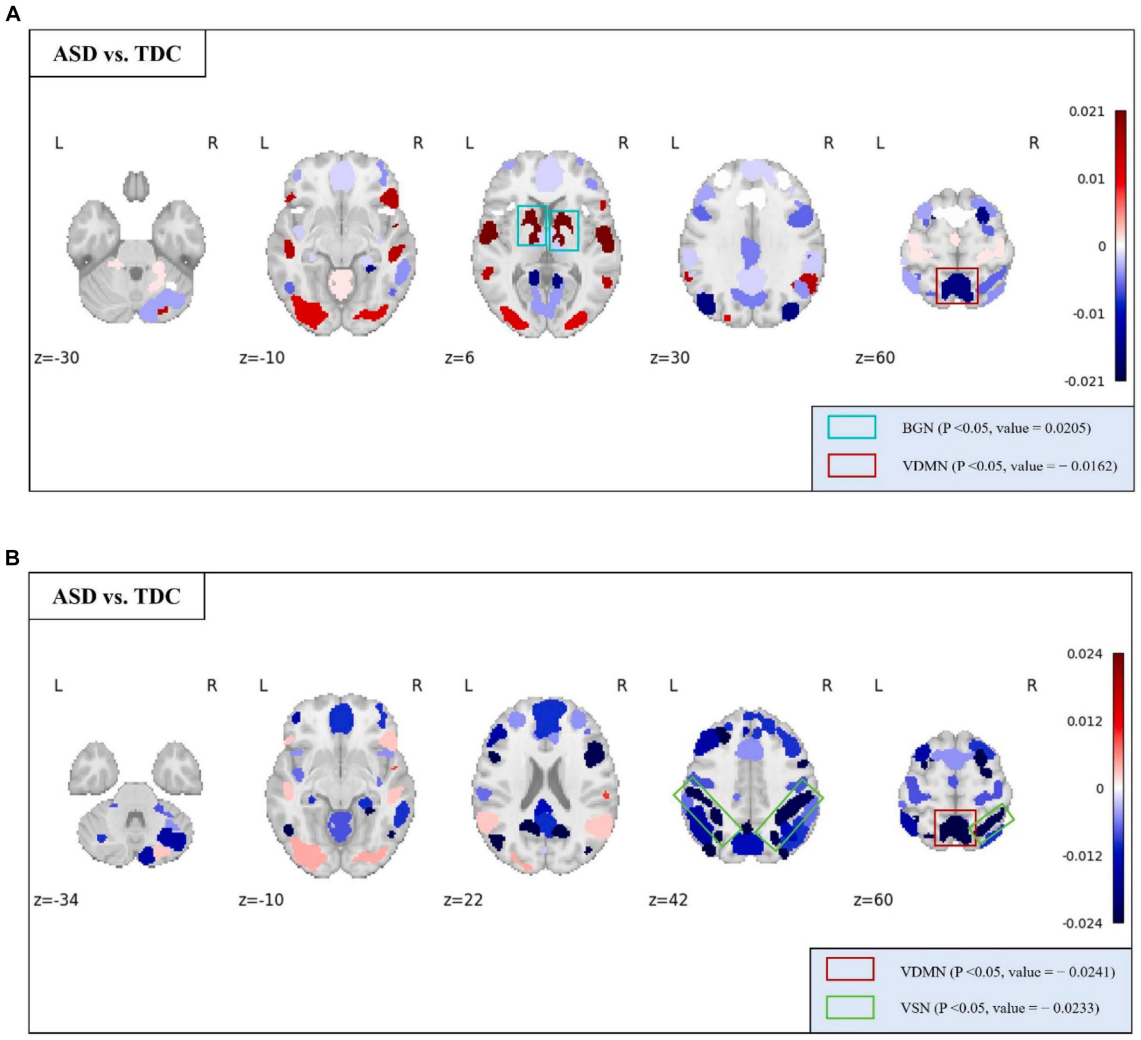
The distribution of differences in group-level average of the temporal centrality and local functionality between the ASD and TDC groups. Colored boxes: Indicate MRSNs with significant differences; P: p-value of independent samples *t*-test; Value: Difference between ASD and TDC. **(A)** Temporal centrality; **(B)** Local functionality.

At the MRSN scale, we found that there were significant differences in temporal centrality for BGN and VDMN between the two groups. The *t*-test results for temporal centrality and local functionality between the ASD patients and the TDC group are shown in Figure 7. The abnormal changes in temporal centrality and local functionality in GM-related MRSNs in ASD patients compared to the TDC group are illustrated in Figure 8. Specifically, temporal centrality in BGN (A/T: 0.1535/0.1330) was significantly higher in the ASD patients compared to the control group, while temporal centrality in VDMN (A/T: 0.1356/0.1518) was significantly higher in the TDC group compared to the ASD patients. Moreover, significant differences in local functionality were observed between VDMN (A/T: 0.1607/0.1848) and VSN (A/T: 0.1299/0.1532) in the two groups, with the TDC group exhibiting significantly higher local functionality than the ASD patients. However, there were no significant differences in temporal stability and joint functionality at the MRSN scale between the two groups. Please refer to Supplementary Table S3 for the group-level average results of ASD and TDC groups for all MRSNs.

### 3.5 Individual differences

At the whole-brain level, the ICC values for all rich-club metrics were lower than the corresponding benchmarks, with temporal centrality and stability ICCs significantly higher than local functionality and joint functionality.

**Figure 9:**
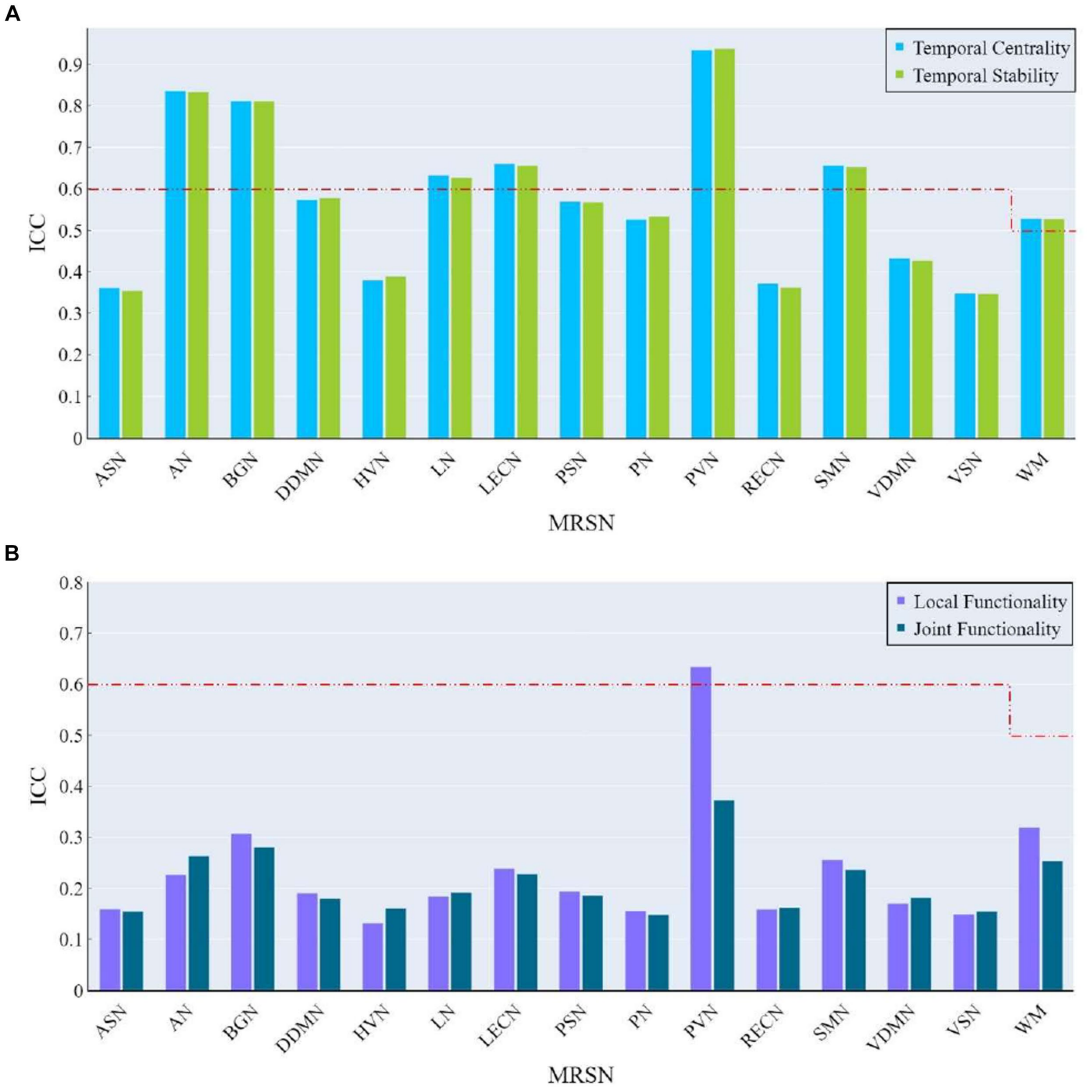
The ICC of rich-club metrics at MRSN scale. **(A)** The ICC of temporal centrality and stability at MRSN scale. **(B)** The ICC of local functionality and joint functionality at the MRSN scale.

At the MRSN level, the ICC of temporal centrality and stability for AN, BGN, LN, LECN, PVN, SMN, and WM were higher than the corresponding benchmarks, with AN, BGN and PVN identified as significantly individual difference regions (Figure 9A). For rich-club metrics related to functional interaction, only PVN exhibited ICC values higher than the benchmarks in local functionality (Figure 9B). While the WM regions did not exhibit results surpassing the benchmarks for local functionality and joint functionality at the MRSN scale, the right hippocampus (LF/JF: 0.6269/0.5749) and left hippocampus (LF/JF: 0.6650/0.5894) within the Cingulum demonstrated ICC exceeding the specified benchmarks in these two rich-club metrics. Table 4 provides a detailed presentation of all MRSN information that exceeds the respective benchmarks. Additionally, individual difference analysis was applied in the validation experiment of methods robustness. The results indicate a high consistency between the individual differences analysis based on XTRACT WM atlases and those based on JHU DTI-based WM atlases. For ICC results using the XTRACT WM atlas and discussions on robustness validation, please refer to Supplementary Figure S3.

**Table 4:** ICC analysis results of MRSNs.

Index	Rich-club metric			Label
	Temporal centrality	Temporal stability	Local functionality	
2	0.8360	0.8334	–	Auditory
3	0.8117	0.8114	–	Basal Ganglia
6	0.6326	0.6269	–	Language
7	0.6597	0.6561	–	LECN
10	0.9345	0.9375	0.6340	Prim Visual
12	0.6563	0.6519	–	Sensorimotor
15	0.5278	0.5273	–	WM

LECN, left executive control network; WM, white matter.

## 4 Discussion

In previous studies, the analysis of brain FCNs and rich-club organization has predominantly focused on the examination of static single-layer networks. However, traditional static single-layer FCN fail to capture the spatio-temporal dynamic changes in the rich-club organization of the brain network. To overcome these limitations, we propose a novel approach to construct whole-brain spatio-temporal multilayer FCNs. Additionally, we introduce four rich-club metrics to characterize the spatio-temporal variations in the rich-club structure of this multilayer FCNs: temporal centrality, temporal stability, local functionality, and joint functionality. Temporal centrality and stability are defined to identify core brain nodes over the entire temporal duration, respectively considering time frequency and duration as essential criteria for core node designation. Local functionality and joint functionality regard core nodes as central hubs for functional interactions within brain network regions, quantifying the propensity of internal and external functional interactions in the brain functional network.

In this work, to validate the effectiveness and interpretability of the proposed multilayer FCNs and rich-club metrics, we preliminary employed the ConvLSTM model to demonstrate the efficacy of the proposed multilayer FCNs in extracting brain network structural features. Subsequently, we applied the proposed methods to three independent analytical tasks: individual differences analysis, male–female gender differences analysis, and analysis of abnormality in patients with ASD. The results indicate that the proposed spatio-temporal multilayer FCNs and four rich-club metrics demonstrate statistical significance and interpretability in the neuroscience.

In the analysis of male–female gender differences, the four rich-club metrics of exhibit diverse differences across different MRSNs, revealing some valuable findings. Gender differences in AN, as previously established in research (Don et al., 1993), were confirmed in our study. Specifically, all four rich-club metrics of AN displayed significant differences, with the male group exhibiting higher average values than the female group. Higher values of temporal centrality and temporal stability indicate a more stable rich-club structure, which could potentially explain why females tend to be more sensitive than males in emotion recognition from voices (Mao et al., 2017). The temporal centrality, local functionality, and joint functionality of the ASN also exhibited significant differences, with the male group displaying significantly higher values compared to the female group. This suggests a stronger intra-network and inter-network FC within the ASN in the male group. In line with this, previous research has demonstrated that males exhibit stronger FC within the salience network (Filippi et al., 2013). Additionally, Zilles and colleagues found that males exhibit stronger activation of visual spatial working memory during tasks (Zilles et al., 2016). In our study, both the local functionality and joint functionality of the VSN exhibited significantly higher values in males compared to females. The stronger functional interaction capability of the VSN in males may reflect their advantage in visual spatial working memory ability.

The temporal centrality and local functionality of the multi-layer FCN revealed distinctive features in certain MRSNs among ASD patients. Specifically, significant differences between ASD patients and the TDC group were observed in the temporal centrality and local functionality of the VDMN, with lower mean values observed in ASD patients. As defined previously, lower values of temporal centrality and local functionality indicate lower FC density and decreased frequency of internal functional interactions within the network. Previous study has reported a significant reduction in FC within the DMN in ASD patient (Floris et al., 2016), and our findings support this conclusion. The DMN is a critical network involved in cognitive and memory functions, and our findings may contribute to understanding the impaired social and communication abilities in ASD patients. It is noteworthy that the temporal centrality of the BGN exhibited significant differences, with ASD patients showing higher average values compared to TDC. Clinical symptoms of ASD include behavioral features such as repetitive and restrictive behaviors (Fakhoury, 2015), with the BGN considered to play a crucial regulatory role in action selection (Chakravarthy et al., 2010; Calderoni et al., 2014). A previous study suggested that activation of the basal ganglia in ASD patients leads to increased synchrony between cortical areas, indicating a weakened ability of the basal ganglia to filter brain signals (Prat et al., 2016). The increased anomaly in temporal centrality within the BGN suggests that its internal nodes maintain denser FC over time, indicating that the BGN processes functional interaction information indiscriminately. This inability to prioritize and filter important functional interaction information may contribute to impaired behavioral selection abilities in ASD patients.

The temporal centrality and stability of the multilayer network effectively capture individual differences, introducing new insights into the study of individual differences in functional networks from WM perspective. Based on the ICC results, the high consistency of temporal centrality and stability suggests robust test–retest reliability of the topological temporal characteristics of specific functional networks across different individuals. Previous studies reported individual differences in the activation levels of the left and right superior temporal gyrus, belonging to the AN, during tasks related to hearing and reading comprehension (Yeatman et al., 2010). The results indicate that such individual differences may not only be present during task-related brain activation but also the resting state. The results also present that the temporal centrality and stability of the BGN and the PVN significantly reflect individual differences. The functional activation of BGN has been linked to individual motor proficiency (Tomassini et al., 2011), and PVN plays a crucial role in forming individual differences in cognition and behavior (Kanai and Rees, 2011). Our findings corroborate these studies from the perspective of rich-club organization. It is noteworthy that the topological temporal characteristics of WM may reflect individual differences, possibly due to WM providing essential anatomical connectivity for normal cognitive functioning, leading to systematic associations between individual differences in WM and different functional networks.

Interestingly, the WM subregion hippocampus reflects individual differences in both function-related rich-club metrics. Probably the most well recognized function of the hippocampus is its role in memory and learning, and damage to both hippocampi can result in retrograde amnesia, an inability to form and retain memories of past events. The individual differences of hippocampus may provide new insights into the biological mechanisms of neurocognitive disorders such as AD and retrograde amnesia, as well as contribute to individual clinical diagnoses.

In summary, this work has introduced a method for constructing whole-brain multilayer FCNs and defined four rich-club metrics. These approaches enable the exploration of whole-brain FCNs topological characteristics from both temporal and spatial perspectives, demonstrating exceptional performance in various tasks related to significant difference recognition. In the analysis of gender differences and anomalies in ASD patients, some MRSNs exhibit significant inter-group differences in temporal centrality and local functionality. These findings align with previous neuroscience research on cognition and behavior. Additionally, in the analysis of individual differences, the results suggest that the topological characteristics of the WM region may exhibit individual variability, and the proposed FCNs model and rich-club metrics demonstrate robustness across different WM templates. In summary, the proposed method may provide an effective approach for studying WM-related brain networks.

Finally, it is imperative to acknowledge certain limitations within this study. The method utilized for defining core nodes involved selecting the top 15 nodes based on normalized degree within each network layer. However, this approach does not consider the inter-layer coupling existing between diverse FCN layers. To fully exploit the rich information embedded in multilayer FCNs, a more optimal method for defining core nodes will be pursued in our future work. The pathological feature of WM abnormalities in ASD patients has been extensively validated (McGrath et al., 2013; Zhou et al., 2014), yet our study did not exhibit significant differences in this regard. One possible reason is that the architecture of the multi-layer FCN focuses more on functional interactions between WM and GM, neglecting internal functional connections within the WM. This oversight may result in a lack of information regarding white matter functional interactions. We aim to address this limitation in future research endeavors. Additionally, the robustness of the proposed method to functional parcellations of the GM template is difficult to verify. This is due to the requirement for predefined correspondences between “functional networks-subregions” in the gray matter template to describe the functional interactions between and within networks in the first and second layers of the multilayer network. However, publicly available GM templates meeting these criteria are limited. Therefore, a potential direction for model development to address the limitations of predefined GM templates could be functional brain region segmentation based on data.

## 5 Conclusion

This study proposed a novel method for constructing whole-brain spatio-temporal multilayer FCNs and further incorporates four rich-club metrics to analyze the dynamic topological characteristics of brain networks. By integrating graph theory, hypergraph theory, and the sliding time window method, the proposed method effectively captures both low-order and high-order associations among functional brain regions and their temporal dynamics. The experimental results align with previous research while revealing innovative findings. For instance, WM demonstrates the capacity to reflect individual differences, and the observed rigidity in the topological structure of the BGN potentially explains repetitive or restrictive behaviors in patients with ASD. The research findings indicate that the proposed method offers a novel perspective for the construction of whole-brain FCNs and the interpretability of their topological structures, contributing to more reliable biomarkers in the study of brain network differences.

## Data availability statement

The original contributions presented in the study are included in the article/Supplementary material, further inquiries can be directed to the corresponding authors.

## Ethics statement

Ethical approval was not required for the studies involving humans because the local legislation and institutional requirements. The studies were conducted in accordance with the local legislation and institutional requirements. The participants provided their written informed consent to participate in this study.

## Author contributions

JZ: Conceptualization, Formal analysis, Methodology, Writing – original draft. YC: Conceptualization, Methodology, Writing – original draft. XW: Data curation, Supervision, Writing – review & editing. XL: Investigation, Writing – review & editing. YF: Supervision, Writing – review & editing. ZY: Project administration, Writing – review & editing.

## Footnotes

^1^ http://restfmri.net/forum/DPABI
