# Supplementary material for: Rich-club organization of whole-brain spatio-temporal multilayer functional connectivity networks
Source: Front Neurosci. 2024 May 24;18:1405734. doi: 10.3389/fnins.2024.1405734 (PMC11157044; doi:10.3389/fnins.2024.1405734)
Supplement: Supplementary file 1 [file Data_Sheet_1.docx]

Supplementary Material

# Supplementary and Tables

**Table S1.** Details of the ROIs included in the 14 MRSNs.

| **MRSN** | **ROI Index** | **Number** |
| --- | --- | --- |
| ASN | 1, 2, 3, 4, 5, 6, 7 | 7 |
| AN | 8, 9, 10 | 3 |
| BGN | 11, 12, 13, 14, 15 | 5 |
| DDMN | 16, 17, 18, 19, 20, 21, 22, 23, 24 | 9 |
| HVN | 25, 26 | 2 |
| LN | 27, 28, 29, 30, 31, 32, 33 | 7 |
| LECN | 34, 35, 36, 37, 38, 39 | 6 |
| PSN | 40, 41, 42, 43, 44, 45, 46, 47, 48, 49, 50, 51 | 12 |
| PN | 52, 53, 54, 55 | 4 |
| PVN | 56, 57 | 2 |
| RECN | 58, 59, 60, 61, 62, 63 | 6 |
| SMN | 64, 65, 66, 67, 68, 69 | 6 |
| VDMN | 70, 71, 72, 73, 74, 75, 76, 77, 78, 79 | 10 |
| VSN | 80, 81, 82, 83, 84, 85, 86, 87, 88, 89, 90 | 11 |

Abbreviations: ASN, anterior salience network; AN, auditory network; BGN, basal ganglia network; DDMN, dorsal default mode network; HVN, higher visual network; LN, language network; LECN, left executive control network; PSN, posterior salience network; PN, precuneus network; PVN, primary visual network; RECN, right executive control network; SMN, sensorimotor network; VDMN, ventral default mode network; VSN, visuospatial network.

**Table S2.** The group-level average of rich-club metrics for male group and female group at the MRSN scale.

| **MRSN** | **Rich-club Metric (Male/Female)** | | | |
| --- | --- | --- | --- | --- |
|  | Temporal Centrality | Temporal Stability | Local Functionality | Joint Functionality |
| ASN | **0.2476/0.2228** | 0.2028/0.1907 | **0.3826/0.3427** | **0.2822/0.2575** |
| AN | **0.2372/0.1739** | **0.2211/0.1881** | **0.1765/0.1580** | **0.2418/0.2166** |
| BGN | 0.0266/0.0340 | 0.0381/0.0437 | 0.0987/0.0946 | 0.1589/0.1497 |
| DDMN | 0.1027/0.1118 | **0.1035/0.1164** | 0.2155/0.1999 | 0.2079/0.1952 |
| HVN | 0.3314/0.3511 | 0.3126/0.3158 | 0.3186/0.2903 | 0.3275/0.3029 |
| LN | 0.1469/0.1563 | 0.1541/0.1644 | 0.2995/0.2750 | **0.2481/0.2303** |
| LECN | 0.1931/0.1832 | 0.1953/0.1945 | 0.2996/0.2761 | 0.2567/0.2370 |
| PSN | 0.0857/0.0909 | 0.0928/0.0934 | 0.2079/0.1957 | 0.2047/0.1922 |
| PN | 0.2250/0.2314 | 0.2095/0.2086 | **0.3271/0.3066** | 0.2831/0.2665 |
| PVN | 0.1674/0.1680 | 0.1600/0.1636 | 0.0149/0.0207 | 0.1794/0.1711 |
| RECN | 0.2407/0.2443 | 0.2141/0.2203 | 0.3672/0.3456 | 0.2788/0.2618 |
| SMN | 0.1660/0.1514 | 0.1611/0.1534 | 0.2032/0.1909 | 0.2194/0.2043 |
| VDMN | 0.1911/0.1972 | 0.1760/0.1823 | 0.3468/0.3375 | 0.2595/0.2467 |
| VSN | 0.1513/0.1490 | 0.1561/0.1543 | **0.3684/0.3313** | **0.2633/0.2424** |
| WM | 0.0077/0.0106 | 0.0118/0.0156 | 0.0509/0.0532 | 0.1125/0.1115 |

Bold font indicates significant differences in rich-club metrics for this MRSN. WM, white matter.

**Table S3.** The group-level average of rich-club metrics for ASD group and TDC group at the MRSN scale.

| **MRSN** | **Rich-club Metric (ASD/TDC)** | | | |
| --- | --- | --- | --- | --- |
|  | Temporal Centrality | Temporal Stability | Local Functionality | Joint Functionality |
| ASN | 0.1561/0.1562 | 0.1735/0.1786 | 0.1735/0.1786 | 0.1523/0.1549 |
| AN | 0.1519/0.1315 | 0.1391/0.1298 | 0.1391/0.1298 | 0.1519/0.1499 |
| BGN | **0.1535/0.1330** | 0.1194/0.1215 | 0.1194/0.1215 | 0.1397/0.1424 |
| DDMN | 0.1156/0.1173 | 0.1353/0.1454 | 0.1353/0.1454 | 0.1321/0.1382 |
| HVN | 0.1593/0.1474 | 0.1503/0.1461 | 0.1503/0.1461 | 0.1629/0.1625 |
| LN | 0.1173/0.1020 | 0.1299/0.1272 | 0.1299/0.1272 | 0.1315/0.1310 |
| LECN | 0.1239/0.1282 | 0.1255/0.1354 | 0.1255/0.1354 | 0.1332/0.1414 |
| PSN | 0.1041/0.1062 | 0.1391/0.1461 | 0.1391/0.1461 | 0.1346/0.1404 |
| PN | 0.1638/0.1690 | 0.1628/0.1754 | 0.1628/0.1754 | 0.1538/0.1618 |
| PVN | 0.1034/0.1075 | 0.0683/0.0709 | 0.0683/0.0709 | 0.1344/0.1405 |
| RECN | 0.1760/0.1796 | 0.1885/0.2038 | 0.1885/0.2038 | 0.1610/0.1698 |
| SMN | 0.1194/0.1183 | 0.1474/0.1555 | 0.1474/0.1555 | 0.1432/0.1481 |
| VDMN | **0.1356/0.1518** | 0.1607/0.1848 | **0.1607/0.1848** | 0.1409/0.1517 |
| VSN | 0.1122/0.1184 | 0.1299/0.1532 | **0.1299/0.1532** | 0.1268/0.1397 |
| WM | 0.0685/0.0675 | 0.0974/0.0938 | 0.0974/0.0938 | 0.1153/0.1164 |

Bold font indicates significant differences in rich-club metrics for this MRSN.


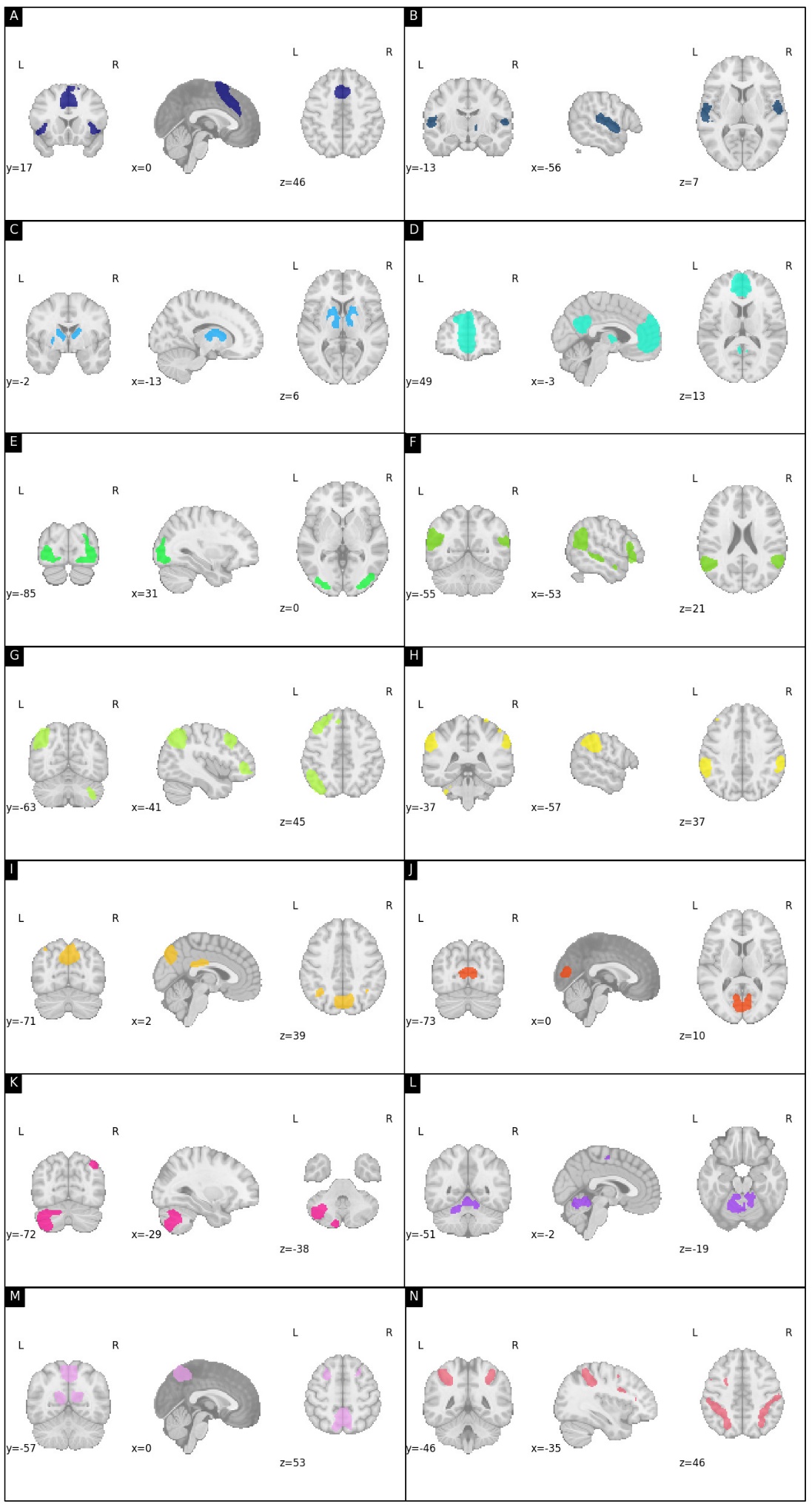


**Figure S1.** The individual spatial distribution of each of the 14 macroscale resting-state networks (MRSNs) across the brain (Shirer et al., 2012). (A) ASN, (B) AN, (C) BGN, (D) DDMN, (E) HVN, (F) LN, (G) LECN, (H) PSN, (I) PN, (J) PVN, (K) RECN, (L) SMN, (M) VDMN, (N) VSN.

**
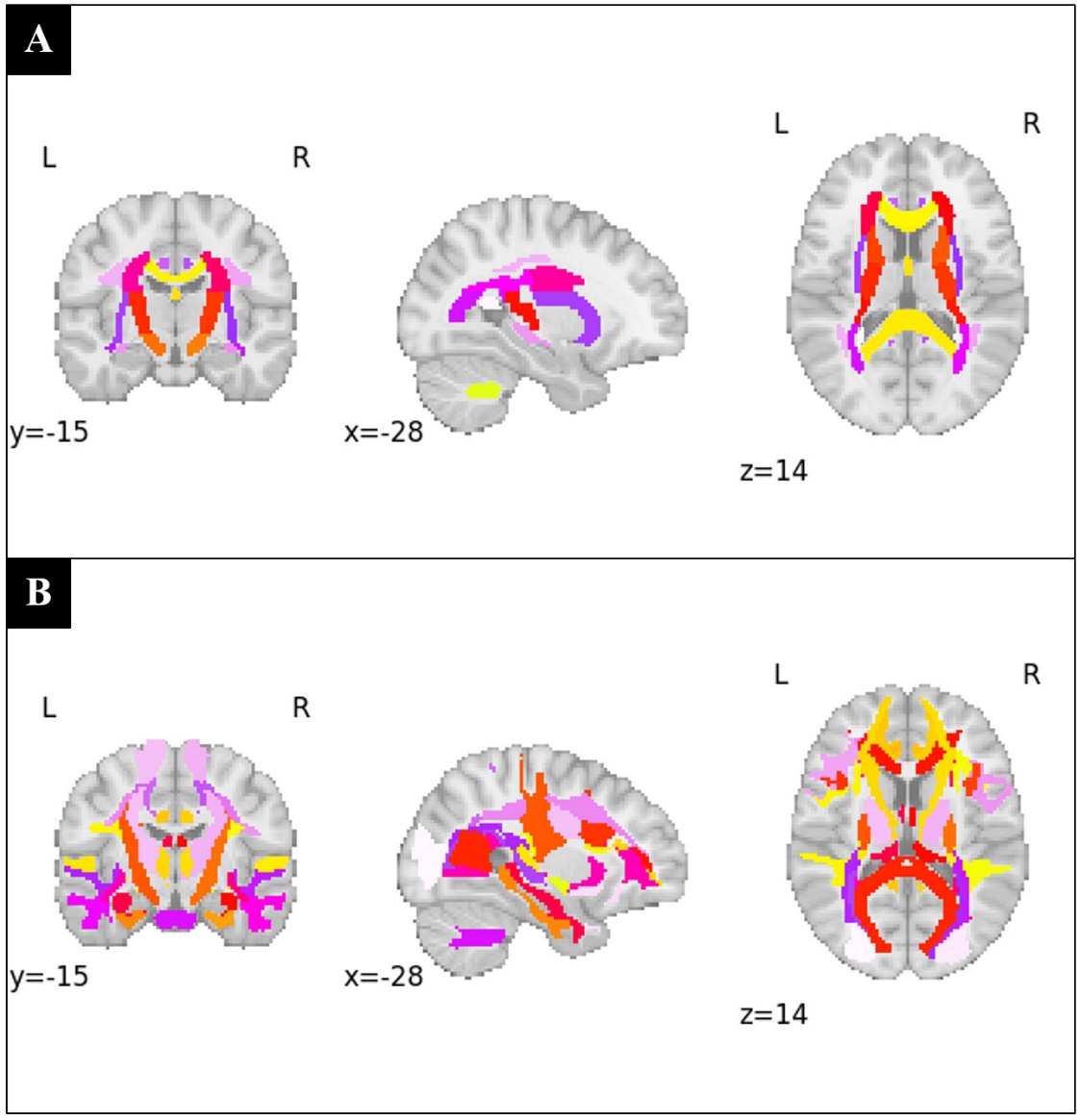
**

**Figure S2.** A brief schematic diagram of the white matter tracts included in the two white matter templates. (A) JHU DTI-based WM atlases (Mori et al., 2008), including a total of 48 probabilistic WM tracts. (B) XTRACT HCP Probabilistic Tract Atlases (Warrington et al., 2020), including a total of 42 probabilistic WM tracts.

Considering the structure of the proposed whole-brain spatio-temporal multilayer functional connectivity network, we employed different WM templates to validate the robustness of the proposed method. Specifically, we utilized the XTRACT HCP Probabilistic Tract Atlases instead of the JHU DTI-based WM atlases to construct the network model and applied the multilayer network in individual difference analysis experiments. The ICC results based on the XTRACT atlas template are depicted in Figure S3.

Combining the ICC results from the two different white matter templates, it can be observed that the proposed method exhibits good robustness when facing different white matter templates. In terms of time-related rich-club metrics, including AN, BGN, DDMN, PSN, PVN, and WM, the ICC values are all above 0.6, showing high consistency with previous research results. This consistency is also evident in the function-related rich-club metrics, i.e. only the ICC of PVN's local functionality exceeding 0.6. In summary, we believe that the proposed method demonstrates good robustness across different WM templates.

**(A)**

**
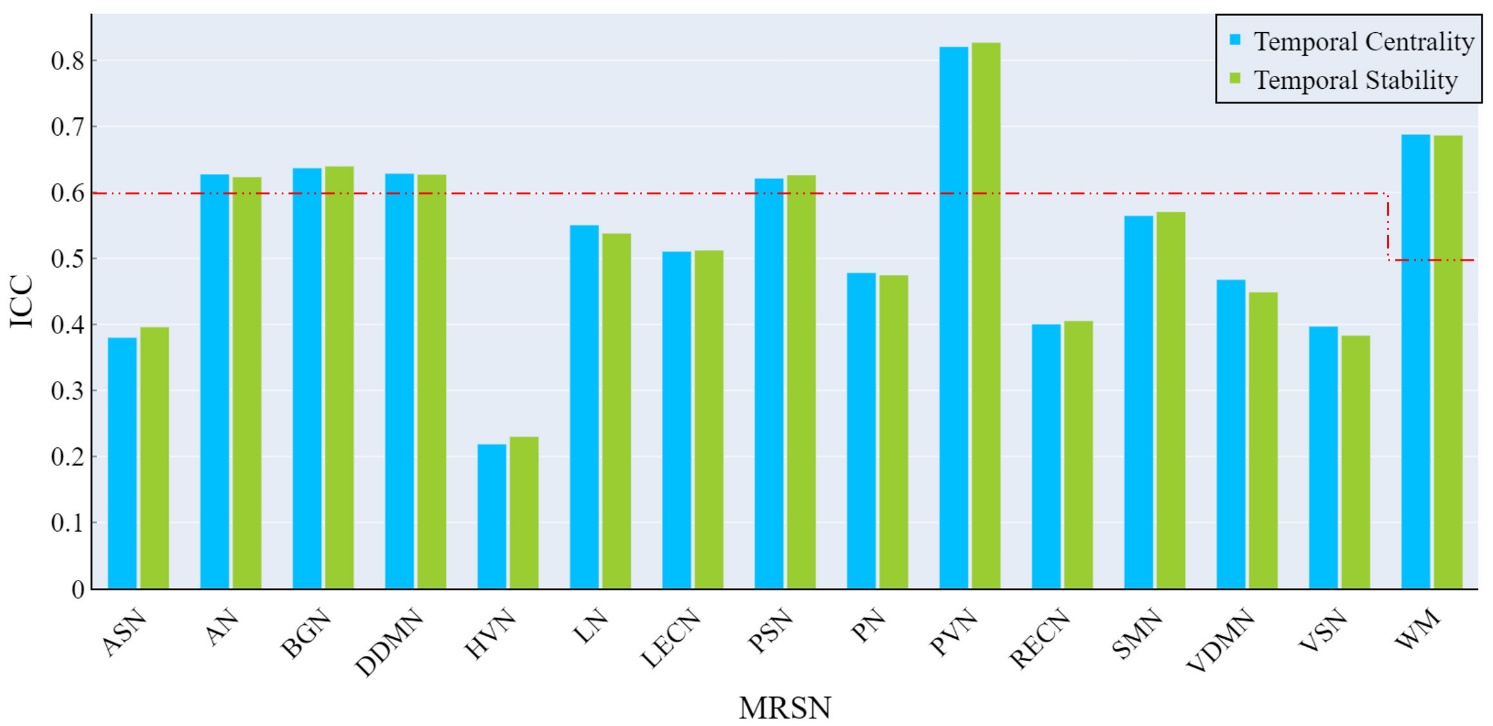
**

**(B)**

**
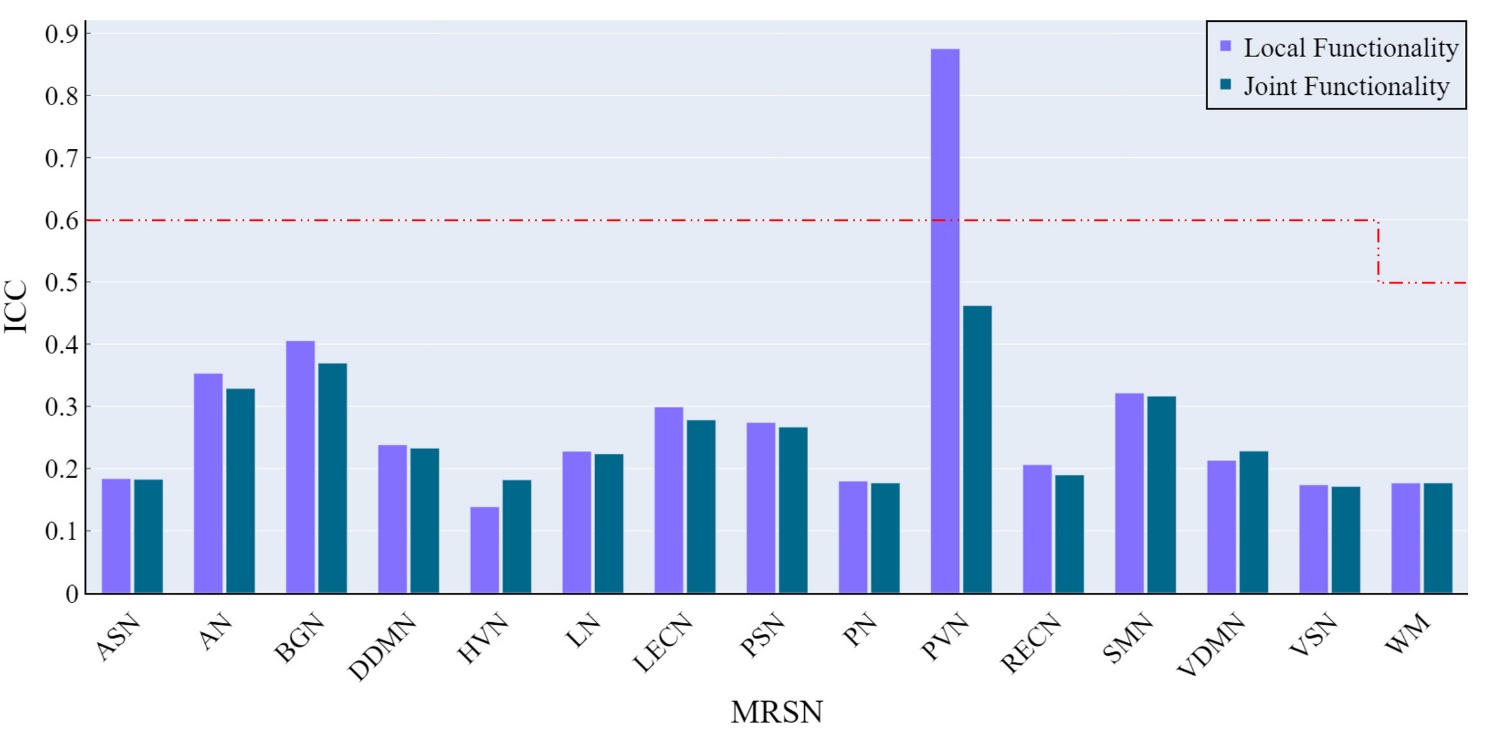
**

**Figure S3.** The ICC of rich-club metrics at MRSN scale (using XTRACT HCP Probabilistic Tract Atlases). (A) The ICC of temporal centrality and stability at MRSN scale. (B) The ICC of local functionality and joint functionality at the MRSN scale.

# References

Mori, S., Oishi, K., Jiang, H., Jiang, L., Li, X., Akhter, K., et al. (2008). Stereotaxic white matter atlas based on diffusion tensor imaging in an ICBM template. *Neuroimage* 40, 570–582. doi: 10.1016/j.neuroimage.2007.12.035

Shirer, W. R., Ryali, S., Rykhlevskaia, E., Menon, V., and Greicius, M. D. (2012). Decoding Subject-Driven Cognitive States with Whole-Brain Connectivity Patterns. *Cerebral Cortex* 22, 158–165. doi: 10.1093/cercor/bhr099

Warrington, S., Bryant, K. L., Khrapitchev, A. A., Sallet, J., Charquero-Ballester, M., Douaud, G., et al. (2020). XTRACT - Standardised protocols for automated tractography in the human and macaque brain. *Neuroimage* 217, 116923. doi: 10.1016/j.neuroimage.2020.116923
